# Electricity and Its Application to Dentistry

**Published:** 1887-05

**Authors:** George C. Brown


ARTICLE II.

### ELECTRICITY AND ITS APPLICATION TO DENTISTRY.

BY DR. GEORGE C. BROWN.

Thinking that a brief history of electricity would be of interest to you I have taken the liberty of going back to the earliest recorded accounts, as compiled by W. J. Lancaster, F. C. S., F. R. A. S. The first record he gives relates to 4,500 years ago.

At this remote period of our earth's history the Chinese were acquainted with the power possessed by lodestone of attracting particles of iron. Whether they knew of attraction and repulsion we cannot tell, and nothing is recorded of any further knowledge of magnetism or electricity until some 2,000 years later, or 580 B. C., when Thales, of Miletus, the founder of the Ionic philosophy, discovered that amber (electron), when rubbed, possessed the power of attracting particles of decayed leaves, pieces of straw, etc. Thales believed that amber was an animate body, and in fact regarded the world as a living being and that everything was derived from water.

Again another period of over 2,000 years elapsed without any important fact being recorded, when in A. D. 1576 Robert Norman discovered that when a nicely poised needle was magnetized the north end became apparently heavier, and the south pole had to be weighted to give again a true balance, hence the inclination of the magnetic needle was discovered by Norman. Some twenty-four years later, in A. D. 1600, Dr. Gilbert, of Colchester, wrote a small work in which he describes some new experiments, proving that other bodies besides amber had similar properties under like conditions. Among such substances he enumerates glass, resin, spars, gems, stones, sulphur, etc., and he first gave the terms North and South pole; also a theory accounting for terrestial magnetism. Although Dr. Gilbert's work was received with enthusiasm three-fourths of a century had nearly elapsed before anything worth recording took place, when in 1672 Otto Guericks announced his discovery of a machine consisting of a ball of sulphur, which, when rubbed by the hand, produced sparks of electricity. Three years later, in 1675, Sir Isaac Newton found that a glass globe was preferable to a ball of sulphur, and Sir Isaac also found that by rubbing a flat glass held a short distance above the table small bodies on the table were lifted towards it.

The 17th century closed without any further discovery being reported, and in 1729 Stephen Grey, a pensioner at the Charter-house, after numerous experiments, found that bodies were either conductors or non-conductors of electricity, and he conducted a current through wet string, the first experiment, leading on to the establishing of telegraphs throughout the world. Four years later, in 1733, Dufay discovered that conductors were non-electrics, and that non-conductors were electrics. He also distinguished between positive and negative electricity.

For several years after this period attention was directed to the perfecting of the frictional electric machine, the most important addition being, in 1740, the prime conductor, by Boze, of Wittenberg, and the fixed rubber, by Winkler, of Leipsic. Just after this, in 1746, Muschenbrock and his pupil Cunens, of Leyden, were endeavoring to electrify a flask of water which Cunens held in one hand, while a chain from the machine dipped into the water. After the machine had been turned some time Cunens, in the act of removing the chain, received a severe shock. This was the origin of the Leyden jar, and, as it was the first experiment proving that electricity could be stored, it will rank as the forerunner of all storage apparatus.

One of the greatest discoveries about this time was that in 1749 of Franklin, by which he obtained a parallel between lightning and electricity. After a considerable number of experiments with a large battery of Leyden jars he, in June, 1752, during a storm, flew a kite having metallic points; with this, and a key attached to the lower end of the pack-thread and from this a silk cord attached to a tree, he obtained a considerable number of sparks; not until, however, the rain had saturated the thread. From these experiments Franklin invented the lightning conductor.

From Philadelphia we must travel to Pavia, where, in 1775, Volta invented the electrophorus. In 1782 he made the condenser and in 1799 he made his famous voltaic pile. While Volta was at work in experiments in frictional electricity, at the neighboring town of Bologna Galvani was making equally important experiments, but in an opposite direction. Thus, in 1780, Galvani discovered the contraction in a frog's leg when the crural nerve was connected to a metallic support and the electrical machine worked. At every spark he found a consequent contraction of the leg. In 1786 he obtained the same effect by the contact of two dissimilar metals, and after a great number of experiments, in 1791, he published a complete account of them, which led to a considerable amount of correspondence between Galvani and Volta. But the first to explain the true cause of the action in 1792 was Fabroni, of Florence, who enunciated the theory that chemical action was the sole cause. This theory was further confirmed in 1800-7, by Davy, who immediately had made a large number of cells, some 2,000 in all. A full and most interesting account of these cells, and the then remarkable experiments and researches made with them, may be found in the "Baberian Lecturer" of November 20, 1806, and November 19, 1807. In the latter one Davy foreshadows his discovery in 1809 of the electric light, which he obtained from two pencils of charcoal, each one inch long and one-sixteenth inch in diameter. The spark obtained when the points touched was increased to an arc four inches in length by the separation of the carbon points. Afterwards an arc of seven inches was obtained in an exhausted receiver, and so intense was the heat that, Davy tells us, not only were the charcoal points consumed but also the platinum wire to which they were attached was melted and fell down in large globules.

Since that time only seventy-years have elapsed, and it will be quite impossible to chronicle one-tenth of the discoveries and inventions made in this period of time. A discovery of the utmost importance was made in the latter part of the year 1819 by Oersted, the then Secretary of the Royal Society of Copenhagen. He had, in 1813, published a work in which he hinted at a connection between magnetism and electricity, but it was six years later when he made the important discovery that a current of electricity passing through a wire over a magnetic needle caused the needle to deflect. The announcement of this experiment excited the greatest interest among all the philosophers of Europe, and in 1820 Arago invented the electro-magnet. Schweigger, of Halle, also invented the galvanometer, which Ampere first exhibited at a meeting of the Royal Academy of Science, and in the following year, 1821, Faraday discovered electro-magnetic rotation. The most important of the great discoveries of the noble Faraday was that in 1831, when he obtained electricity from the inductive action of terrestial magnetism. In the year following, 1832, Pixii made the first magneto-electric machine, which was improved and added to in 1833 by Sexton, of Philadelphia, who made the bobbins to rotate in place of the rotating magnet of Pixii. The next important improvement was made by Siemens in 1854, in which he wound the wires of the armature longitudinally instead of transversely, as in the old machine. The first time the electric light was used in a public manner was in 1836, when it was used to illustrate the rising sun in "Le Prophet," at the opera in Paris.

My first knowledge of electricity being applied to dentistry was in 1859, when considerable interest was awakened in the profession in its application to the extraction of teeth. Many claimed that the painless extraction of a tooth could be accomplished by passing a current through the tooth at the time of its removal. I cannot do better than to describe the apparatus of one of the advocates of this method. "This apparatus consisted of an electro-magnetic machine with a metallic rod at the end of each cord, the rod at the positive pole to be connected by means of a steel hook; second, an extensor, which is a piece of flexible conducting cord about a foot long, with a metal loop at each end, one very small; third, a conductor, which is composed of a gutta-percha tube eight inches in length, and a piece of copper wire with a small piece of sponge at one end, the wire to pass through the tube and turn into a loop at the other end, drawing the sponge close up to the tube. The battery should be placed conveniently in rear of the chair. The mode of operating is as follows: Extracting a tooth, set the battery in action, place a rod in each hand of the patient, and pass the piston into the helix until the muscles of the wrists begin to contract slightly; note the position of the piston and withdraw it; take a piece of spool thread and pass it through the small loop of the extensor, and tie it into a loop, full large enough to pass over the tooth; cut off the ends, dip the loop into water, and slip it over the tooth, twist the cord until the thread is close to the tooth; take the rod of the positive pole from the patient, let him grasp the other (negative) in both hands, unhook the positive rod and connect with the extensor; the forceps may now be placed in position, and the current introduced by passing the piston slowly into the helix; if the patient winces the least, let the piston remain stationary a few seconds, then pass it still further, slowly and carefully, until the measure is obtained, that was passed through the hands, or is so strong as to border on the disagreeable. The tooth may then be removed deliberately, the slower the better." My own experience was that in a great many cases the shock was more painful than the extraction of the tooth.

The first really practical application of electricity to dentistry was the Bonwill electric mallet, invented by Dr. W. G. A. Bonwill of Philadelphia, February 27, 1867. It was improved upon until 1876, and placed in its present practical shape in 1879. The first three forms had the stroke governed by clock-work ratchet, which would run an hour. Since 1871-72, the automatic brake has been upon the instrument, and has been made very compact. The first electric mallet weighed one pound, and had to be suspended from the ceiling; the last one weighs but five and one-half ounces, and is used in the hand with perfect facility. Of this mallet the report of the "International Electric Exhibition, Section XXIV, Electro-dental apparatus," says: "This section finds presented for its examination, first The Bonwill Electro-Magnetic Mallet. * * The Bonwill Electro-Magnetic Mallet, under the title of 'Historical Exhibit of the Electro-Magnetic Mallet, and the dental and surgical engines, by Dr. W. G. A. Bonwill of Philadelphia, Pa.' We have a most interesting series of ingenious inventions, indicating gradual advances towards a desired end. It seems to us, that to do them and the inventor justice would require more space than is at our command, but we equally feel that from the electro-historical standpoint, it behooves us to state the claims made to the section by the exhibitor. There were, first, that he is the original inventor of the first practical Electro-Magnetic Mallet for the filling of human teeth with gold, *automatically* by power; second, that he is the only one who has placed upon the same any improvements of any value up to the present time."

The transmission of power by electricity through a wire offering but a limited resistance has already been successfully accomplished. Since a dynamo-electric generator may serve also as an efficient electro-motor the invention of a thoroughly successful generator of electricity from mechanical power solved the problem of the recovery of a large per cent. of this power from the electric current at a more or less remote distance. To the electric motor, therefore, we must look for the utilization of this power. In cities and towns where the electric light is used the motor is a power that can be used to great advantage. Its great value consists, First, from its portability, seeing that it need only be connected by means of a pair of thin flexible wires with the main supply cable. Second, from their small weight per horse power developed by them. Third, from the possibility of their being used at a considerable distance from the prime mover. Fourth, from their working to the best advantage when run at a high rate of speed, and so being especially suitable for driving quick moving machinery direct, without any intermediate gearing. But to those of us who cannot make use of the electric cable the battery is the next generator of the electric current that can be made servicable to us. But the subject of batteries is one so broad and far-reaching that I do not propose to go into any description of them. It is sufficient to say that we have before us several of the new and most approved styles that have been placed on the market. I am not prepared to say which is best, as each one has its good points, and the most singular part of it is that this subtle fluid can be generated from so many different elements, combined in such a quantity of ways, each producing the same effect, varying only in quantity. In regard to motors, the "Griscom" is the only one that has been especially adapted to use in the dental profession, but others are coming forward seeking recognition at our hands, among them I will mention the "Diehl" motor, which I have had much pleasure in watching from its conception, until now it claims a place among the things most needed in the dental office.

To say that a good motor adapted to our use would be received with pleasure, is putting it mild. When, by a touch of the key, our engine is driven at a speed of from two to four thousand revolutions a minute; then again, when we have the apparatus, another turn of the key and the electric light breaks forth with the brilliancy of a minature sun, enabling us to examine the mouth and teeth in a manner never conceived of before. Still another move of the key and the invisible fan is set in motion, cooling our over-heated brains and making the patient feel that their comfort is studied as well as our own.

I predict in the near future great results in the field of electricity as applied to our profession, so great, indeed, that were I to put on paper what I believe will be accomplished, I should be called visionary in the extreme. I trust that you will give the different appliances exhibited a fair and full examination, for I take great pleasure in saying that never before has so fine an exhibition of dental appliances been made at any dental society.—*Proceedings of the New Jersey State Dental Society.*

